# Identification of a novel mutation in the *BMPR2* gene in a pulmonary arterial hypertension patient using next‐generation sequencing

**DOI:** 10.1002/jcla.24183

**Published:** 2021-12-24

**Authors:** Xiao Xu, Xin Wang, Guo‐Can Yang, Qi Liu

**Affiliations:** ^1^ Department of Radiology Shaoxing People's Hospital (Shaoxing Hospital, Zhejiang University School of Medicine) Shaoxing China; ^2^ Department of Rheumatology and Immunology Shaoxing People's Hospital (Shaoxing Hospital, Zhejiang University School of Medicine) Shaoxing China; ^3^ Department of Transfusion Shaoxing People's Hospital (Shaoxing Hospital, Zhejiang University School of Medicine) Shaoxing China

**Keywords:** bioinformatics analysis, *BMPR2*, bone morphogenetic protein type II receptor, genetic test, pulmonary arterial hypertension

## Abstract

**Background:**

Pulmonary arterial hypertension (PAH) is a hemodynamic state that is characterized by pulmonary vasoconstriction and vascular remodeling, leading to a continuous increase in mean pulmonary arterial pressure, and eventually right heart failure. Mutations of the bone morphogenetic protein type II receptor (*BMPR2*) gene are the most common genetic cause of PAH.

**Methods:**

A 52‐year‐old woman was admitted to Shaoxing People's Hospital after suffering from a cough for 2 months. In our hospital, the proband got a thorough medical examination and was diagnosed with PAH following genetic testing.

**Results:**

Genetic test showed that the proband carried a novel heterozygous c.1481C>T (p.Ala494Val) mutation in the *BMPR2* gene. The new mutation was initially discovered as a potential pathogenic variant by bioinformatics research, but it needed to be functionally verified.

**Conclusions:**

The novel mutation may be related to the development of the PAH. In addition to general examinations, clinicians must thoroughly examine molecular genetics to provide an accurate diagnosis in the clinic, particularly for rare disorders.

## Graphical abstract

A novel heterozygous mutation in the *BMPR2* gene, c.1481C>T (p.Ala494Val). Bioinformatics analysis suggested the variant disease‐causing, the Pro residue at 494 in the BMPR2 protein was highly conserved, suggested that the *BMPR2* gene mutation is disease‐causing and may be responsible for the phenotypes of pulmonary arterial hypertension.

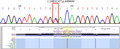

## 1 INTRODUCTION

Pulmonary arterial hypertension (PAH) is a hemodynamic state that is characterized by pulmonary vasoconstriction and vascular remodeling, leading to a continuous increase in mean pulmonary arterial pressure, and eventually right heart failure.^1^, ^2^ Mutation of the bone morphogenetic protein type II receptor (*BMPR2*) gene is the most common genetic cause of PAH.^3^, ^4^ This gene is located on chromosome 2 (2q33‐34) and encodes a member of the bone morphogenetic protein (BMP) receptor family of transmembrane serine/threonine kinases. *BMPR2* is a 190‐kb gene with 13 exons that encodes four conserved domains: extracellular domain, transmembrane domain, kinase domain, and cytoplasmic domain. BMPR2 is a cell‐surface receptor belonging to the superfamily of receptors for ligands of the transforming growth factor TGF‐beta family. This ligand/receptor complex plays an important role in embryogenesis, apoptosis, organ development, cell differentiation, and cell proliferation.

In the present study, we report a 52‐year‐old woman diagnosed with PAH carried a novel heterozygous c.1481C>T (p. Ala494Val) mutation in the *BMPR2* gene. Detailed clinical data and the phenotype‐genotype associated with the disease were delineated.

## 2 MATERIALS AND METHODS

### 2.1 Subjects

All procedures conducted in this study involving human participants were conducted in accordance with the Declaration of Helsinki and following the ethical standards of the Ethical Committee of Shaoxing People's Hospital. Informed consent was obtained from all participants in the study. The healthy people acted as controls.

The proband was a 52‐year‐old woman admitted to our hospital after suffering from a cough for two months. She was given a series of clinical and laboratory tests in Shaoxing people's hospital, including brain magnetic resonance imaging (MRI), magnetic resonance spectroscopy (MRS), next‐generation sequencing (NGS), and so on.

### 2.2 Samples collection

Peripheral blood samples of all participants were collected for the extraction of genomic DNA using a genomic DNA kit (TIANGEN BIOTECH, Beijing, China, DP304) according to the manufacturer's instructions.

### 2.3 Genetics test

Genomic DNA was sequenced by next‐generation sequencing (NGS), which refer to the related studies.^5^, ^6^, ^7^, ^8^ The NGS was performed by Agilent SureSelect Human All Exon V6 kits and Illumina NovaSeq 6000 sequencing platform. The paired‐end reads (PE150) were aligned to a Genome Reference Consortium Human Genome Build 37 (GRCh37)–derived alignment set including decoy sequences using the Burrows‐Wheeler Aligner (BWA). Single nucleotide variants (SNVs), small insertions and deletions (indels), and copy number variants were called with GATK Best Practices. The sequencing data for all samples underwent standard quality control checks. It must be achieved that the average coverage depth is more than 100 X, 90.00% of the target region sequencing depth is greater than 20X, and Q30 is not less than 90%. The pathogenicity of the variants was estimated using the American College of Medical Genetics and Genomics (ACMG) guidelines. Suspected pathogenic variation was verified by Sanger sequencing with specific primers (forward primer: 5′ ‐GAGCATGTTCCGTAATCC‐3′ and reverse primer: 5′ ‐TTGTTGGGTCTCAGTTTC‐3′). This part was conducted by the Joingenome Diagnostics Co., Ltd.

### 2.4 Molecular evolution analysis

The modified MYBPC3 protein was also subjected to bioinformatics analysis utilizing accessible software tools. The software Clustal X1.83 was used to analyze evolution conservation among different paralogs and orthologs. Sequences were obtained from https://www.ncbi.nlm.nih.gov/. In addition, the iterative threading assembly refinement (I‐TASSER) server was used to predict the tertiary structure. STRING database (version 11.0) was used to predict protein‐protein interactions of MYBPC3 protein, and the minimum required interaction score and number were set to 0.700 and 10, respectively. Pfam (https://pfam.xfam.org/) and PredictProtein (http://www.predictprotein.org/) were used to determine aligned protein regions, protein secondary structure, and prediction of mutation function.

## 3 RESULTS

### 3.1 Laboratory examination and diagnosis

The proband was diagnosed as PAH based on clinical assessment, chest radiography, electrocardiography, echocardiography, and genetic testing. Patient clinical characteristics and parameters are shown in Table 1.

**TABLE 1:** Patient clinical characteristics and parameters

Examination item	Test value	Reference value
Clinical		
Age, years	55	
Sex, M(F)	F	
Arterial blood gas analysis		
PH	7.425	7.35–7.45
PO2 (mmHg)	93.1	80.0–100.0
PCO2 (mmHg)	40.0	35.0–45.0
Base excess (mmol/L)	1.2	−3.0–3.0
Alveolar‐arterial oxygen tension difference (mmHg)	14.2↓	15.0–20.0
Standard bicarbonate (mmol/L)	25.3↑	21.3–24.8
Carbon dioxide (mmol/L)	23.1↓	24.0–32.0
P50 (mmHg)	24.4↓	24.8–27.8
Oxyhemoglobin (%)	96.2↑	90.0–95.0
Laboratory parameters		
Cardiolipin antibody IgG (GPLU/ml)	11.9↑	0–10.0
Immunoglobulin G (g/L)	18.4↑	7.00–16.00
Complement C4 (g/L)	0.41↑	0.10–0.40
Rheumatoid factor (IU/ml)	56.54↑	0.00–30.00
Antinuclear antibody	Positive	Negative
Anti‐SSA/Ro−60KD antibody	Positive(++)	Negative
Anti‐SSA/Ro−52KD antibody	Positive(++)	Negative
HLA‐B27	Positive	Negative

### 3.2 Gene detection

**FIGURE 1:**
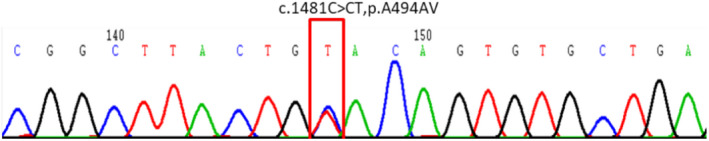
Genome sequencing revealed a heterozygous c.1481C>T mutation in the *BMPR2* gene, which causes amino acid p. Ala494Val. No additional mutation was found

Sanger sequencing identified a novel heterozygous c.1481C>T (p. Ala494Val) mutation in the *BMPR2* gene (Figure 1), whereas the mutation was not found in the controls.

### 3.3 Bioinformatics analysis

**FIGURE 2:**
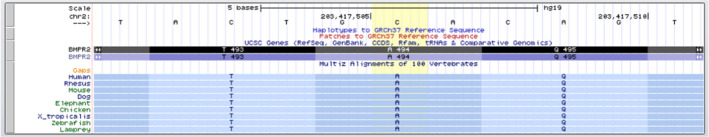
Phylogenetic conservation analysis. Evolution conservation analysis revealed that p.A494 in the *BMPR2* domain was extremely evolutionarily conserved

The predictions of pathogenicity tended to be pathogenic, predictive values of REVEL and CADD were 0.872 and 34, respectively. The conservation analysis indicated that the Pro residue at 494 in the BMPR2 protein was highly conserved across humans, rhesus, mice, dogs, elephants, chickens, x_tropicalis, zebrafish, and lamprey (Figure 2).

**FIGURE 3:**
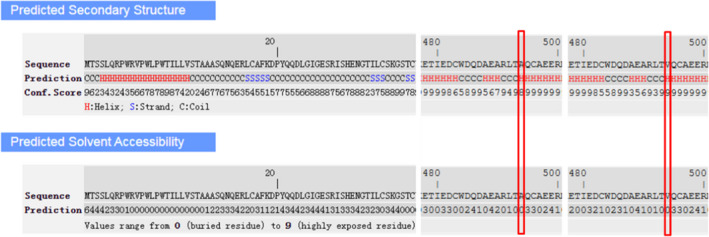
Predicted secondary structure and solvent accessibility predicted by I‐TASSER server. Secondary structures of normal and mutant amino acid are predicted to be helix at position 494 with the respective confidence score of 8 and 9, the confidence ranges 0–9 wherein a higher score indicates a prediction with higher confidence. The solvent accessibility of the sequence is predicted as buried amino acid (range 0–9 wherein a higher value means higher accessibility)

Secondary structure was predicted to be strand by I‐TASSER server with high confidence sore for this variant, solvent accessibility predicted that both normal and mutant amino acids at this position are buried in protein; the accessibility to solvent of each of these amino acids is 4 (Figure 3).

**FIGURE 4:**
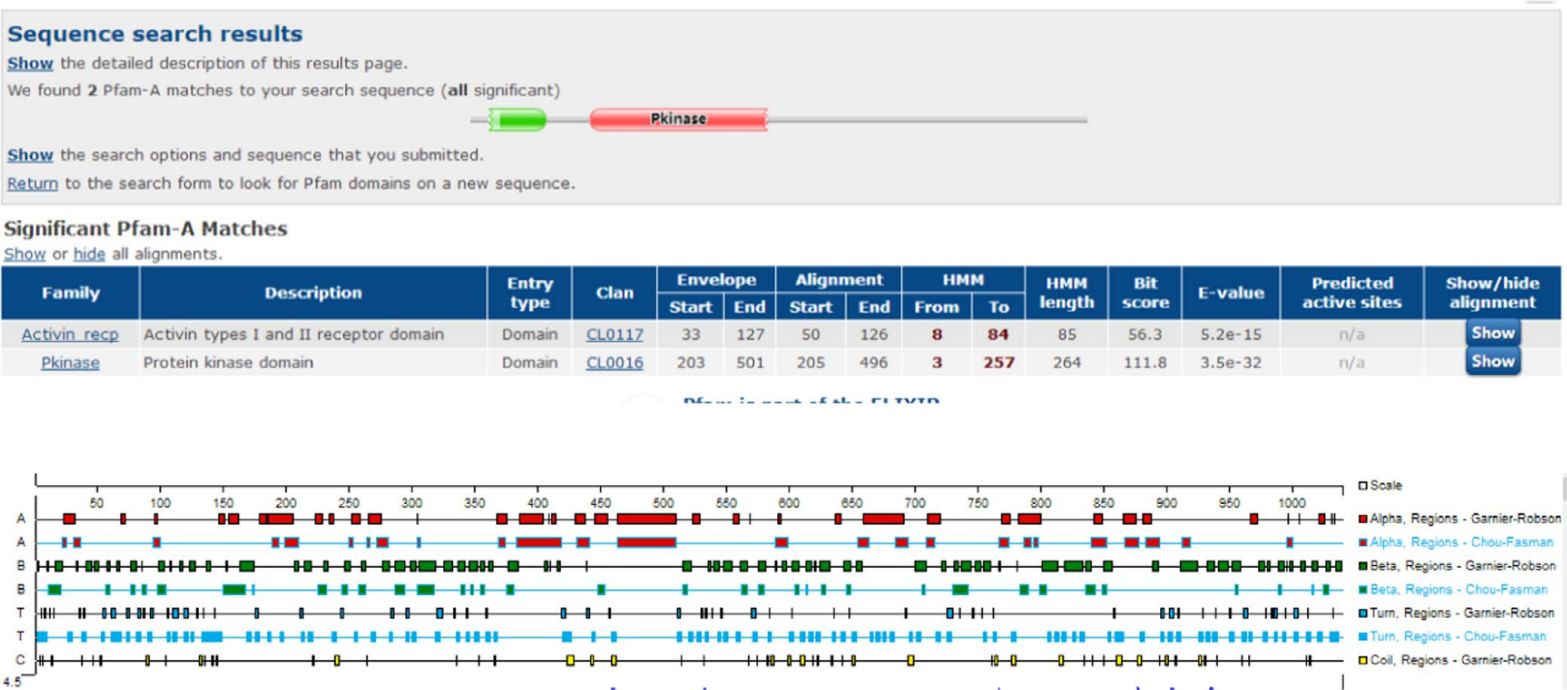
Bioinformatics analysis. The results of protein function prediction, Pfam, reveals the domain contained in the protein sequence (A). Secondary structure simulation showed the relative position of α, β, and random curl (B)

The results of protein function prediction and secondary structure simulation are shown in Figure 4.

**FIGURE 5:**
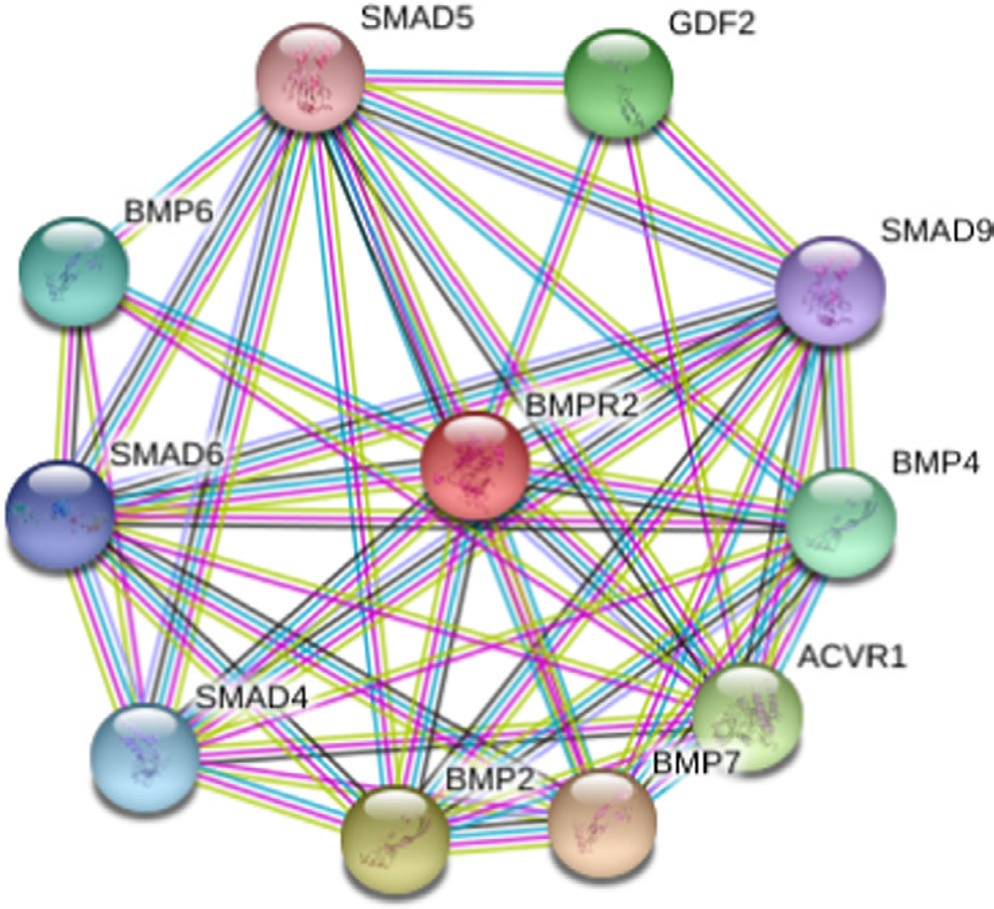
Protein‐protein interaction network of bone morphogenetic protein type II receptor (BMPR2). Predicted functional partners are as follows: BMP7: bone morphogenetic protein 7 (431 aa); BMP2: bone morphogenetic protein 2, induces cartilage and bone formation (396 aa); ACVR1: activin receptor type‐1 (509 aa); GDF2: growth/differentiation factor 2 (429 aa); BMP4: bone morphogenetic protein 4, induces cartilage and bone formation (408 aa); BMP6: bone morphogenetic protein 6, induces cartilage and bone formation (513 aa); SMAD4: mothers against decapentaplegic homolog 4 (552 aa); SMAD6: mothers against decapentaplegic homolog 6 (496 aa); SMAD9: mothers against decapentaplegic homolog 9 (467 aa); and SMAD5: mothers against decapentaplegic homolog 5 (465 aa)

Furthermore, utilizing the STRING database, interactions between BMPR2 and other proteins showed potential implications on bone morphology development. BMP7, BMP2, ACVR1, GDF2, BMP4, BMP6, SMAD4, SMAD6, SMAD9, and SMAD5 are the ten functional partners expected to interact with BMPR2 (Figure 5).

## 4 DISCUSSION

Bone morphogenetic proteins (BMPs) are secreted ligands of the transforming growth factor‐β (TGFβ) family that control embryonic patterning, as well as tissue development and homeostasis.^9^ Mutations in the *BMPR2* gene that induce loss of function are the most common cause of PAH.^10^ *BMPR2*‐associated PAH is an autosomal dominant disease and considered a rare disease, with an estimated incidence of 1–2 per million cases.^11^, ^12^

To date, more than 298 *BMPR2* mutations responsible for 55%–70% of heritable PAH (HPAH) and 11%–40% of idiopathic PAH (IPAH) have been reported.^13^ Despite the impact of *BMPR2* as the main genetic factor for PAH, currently, the specific mechanism of pathogenesis of *BMPR2* in PAH is not fully understood. Normally, BMP signaling activates SMAD proteins that counteract the effects of the TGF‐beta signaling pathway. There is a delicate balance of SMAD signaling among the TGF‐beta receptors. Diminished expression or function of *BMPR2* caused by gene mutations impairs BMP signaling and prevents the appropriate repression of TGF‐beta. Constitutive activation of TGF‐beta signaling drives pulmonary endothelial metabolism and apoptosis while increasing smooth muscle proliferation and cell survival leading to plexiform lesions and increased pulmonary vascular resistance. Now, patients with HPAH and IPAH were recommended to receive genetic counseling and screening for *BMPR2* mutations by the European guidelines, especially to enable predictive genetic testing of relatives.^14^

Patients with PAH who have BMPR2 mutations have been found to appear at an earlier age and have more severe laboratory values than noncarriers, highlighting the necessity of early genetic testing. The unique heterozygous c.1481C>T (p. Ala494Val) mutation in the *BMPR2* gene was initially identified as a potentially pathogenic variant by bioinformatics analysis, but it still needs to be functionally verified.

## 5 CONCLUSIONS

In conclusion, a unique heterozygous c.1481C>T (p. Ala494Val) mutation in the *BMPR2* gene was discovered in a patient with pulmonary arterial hypertension, which appears to be linked to BMP malfunction. However, more research is needed to fully understand and elucidate the underlying mechanism.

## CONFLICT OF INTEREST

None to declare.

## Data Availability

Data sharing is not applicable to this article as no new data were created or analyzed in this study.

## References

[1] Callejo M , Barbera JA , Duarte J , Perez‐Vizcaino F . Impact of nutrition on pulmonary arterial hypertension. Nutrients. 2020;12(1):169. 10.3390/nu12010169 PMC7019983 31936113

[2] Pan T , Zhang L , Miao K , Wang Y . A crucial role of endoplasmic reticulum stress in cellular responses during pulmonary arterial hypertension. Am J Transl Res. 2020;12(5):1481‐1490. 32509157 PMC7269988

[3] Ye F , Jiang W , Lin W , et al. A novel BMPR2 mutation in a patient with heritable pulmonary arterial hypertension and suspected hereditary hemorrhagic telangiectasia: a case report. Medicine (Baltimore). 2020;99(31):e21342. 32756122 10.1097/MD.0000000000021342 PMC7402743

[4] Oriaku I , LeSieur MN , Nichols WC , Barrios R , Elliott CG , Frost A . A novel BMPR2 mutation with widely disparate heritable pulmonary arterial hypertension clinical phenotype. Pulm Circ. 2020;10(3):2045894020931315. 32547734 10.1177/2045894020931315 PMC7273341

[5] Liu D , Morrell NW . Genetics and the molecular pathogenesis of pulmonary arterial hypertension. Curr Hypertens Rep. 2013;15(6):632‐637. 24078385 10.1007/s11906-013-0393-9

[6] Zhang R , Chen S , Han P , et al. Whole exome sequencing identified a homozygous novel variant in CEP290 gene causes Meckel syndrome. J Cell Mol Med. 2020;24(2):1906‐1916. 31840411 10.1111/jcmm.14887 PMC6991682

[7] Dai Y , Liang S , Dong X , et al. Whole exome sequencing identified a novel DAG1 mutation in a patient with rare, mild and late age of onset muscular dystrophy‐dystroglycanopathy. J Cell Mol Med. 2019;23(2):811‐818. 30450679 10.1111/jcmm.13979 PMC6349151

[8] Zheng Y , Xu J , Liang S , Lin D , Banerjee S . Whole exome sequencing identified a novel heterozygous mutation in HMBS gene in a Chinese patient with acute intermittent porphyria with rare type of mild anemia. Front Genet. 2018;9:129. 29731767 10.3389/fgene.2018.00129 PMC5920022

[9] Yang P , Troncone L , Augur ZM , Kim SSJ , McNeil ME , Yu PB . The role of bone morphogenetic protein signaling in vascular calcification. Bone. 2020;141:115542. 32736145 10.1016/j.bone.2020.115542 PMC8185454

[10] Chaikuad A , Thangaratnarajah C , von Delft F , Bullock AN . Structural consequences of BMPR2 kinase domain mutations causing pulmonary arterial hypertension. Sci Rep. 2019;9(1):18351. 31797984 10.1038/s41598-019-54830-7 PMC6892941

[11] Momose Y , Aimi Y , Hirayama T , et al. De novo mutations in the BMPR2 gene in patients with heritable pulmonary arterial hypertension. Ann Hum Genet. 2015;79(2):85‐91. 25612240 10.1111/ahg.12096

[12] Girerd B , Lau E , Montani D , Humbert M . Genetics of pulmonary hypertension in the clinic. Curr Opin Pulm Med. 2017;23(5):386‐391. 28661905 10.1097/MCP.0000000000000414

[13] Frump AL , Datta A , Ghose S , West J , de Caestecker MP . Genotype‐phenotype effects of Bmpr2 mutations on disease severity in mouse models of pulmonary hypertension. Pulm Circ. 2016;6(4):597‐607. 28090303 10.1086/688930 PMC5210048

[14] Ge X , Zhu T , Zhang X , Liu Y , Wang Y , Zhang W . Gender differences in pulmonary arterial hypertension patients with BMPR2 mutation: a meta‐analysis. Respir Res. 2020;21(1):44. 32028950 10.1186/s12931-020-1309-2 PMC7006426

